# CAR-T Cell Therapy Shows Similar Efficacy and Toxicity in Patients With DLBCL Regardless of CNS Involvement

**DOI:** 10.1097/HS9.0000000000000984

**Published:** 2023-11-30

**Authors:** Evgenii Shumilov, Hristo Boyadzhiev, Paolo Mazzeo, Dilara Akhoundova, Michael Daskalakis, Urban Novak, Georg Lenz, Ulrike Bacher, Thomas Pabst

**Affiliations:** 1 Department of Medicine A, Hematology, Oncology and Pneumology, University Hospital Münster (UKM), Germany; 2 Habichtswald Hospital, Kassel, Germany; 3 Clinics of Hematology and Medical Oncology, INDIGHO Laboratory, University Medical Center Göttingen (UMG), Germany; 4 Department of Medical Oncology, Inselspital, University Hospital Bern, University of Bern, Switzerland; 5 Department of Hematology, Inselspital, University Hospital Bern, University of Bern, Switzerland

## Abstract

Efficacy and toxicity of chimeric antigen receptor T (CAR-T) cell therapy in relapsed/refractory (r/r) diffuse large B-cell lymphoma (DLBCL) with central nervous system (CNS) involvement remain understudied. Here we analyzed the outcomes of CAR-T cell therapy in r/r DLBCL patients with CNS involvement and compared them with patients without CNS disease. Retrospective and monocentric comparative analysis of patient cohort with r/r DLBCL treated with CAR-T cell therapy: 15 patients with CNS versus 65 patients without CNS involvement. Overall response rates (80% versus 80%; *P* = 1.0), progression-free survival (*P* = 0.157), and overall survival (*P* = 0.393) were comparable for both cohorts. The frequency of cytokine release syndrome was comparable in the CNS and non-CNS cohorts; 93% versus 80%; *P* = 1.0. Numerically, immune effector-cell–associated neurotoxicity syndrome (all grades) was more frequent in patients with CNS manifestation (53% versus 29%; *P* = 0.063), although no grade 4 events were documented. Our study suggests that CAR-T cell therapy is effective and feasible in patients with r/r DLBCL and CNS manifestation.

## Graphical abstract

**Figure:**
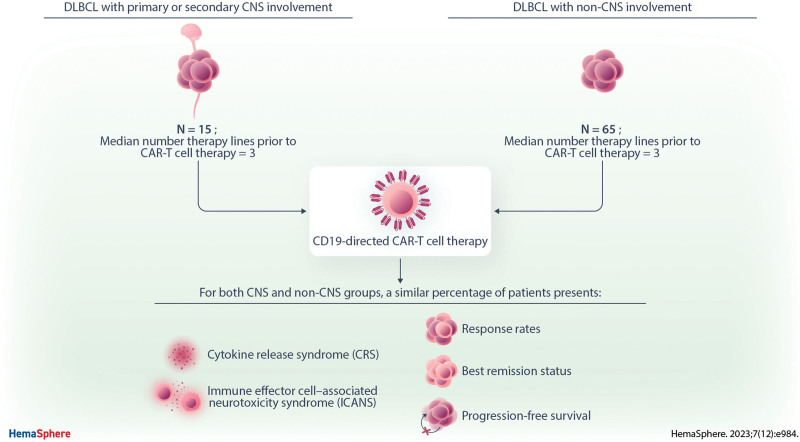

## INTRODUCTION

Relapsed/refractory (r/r) diffuse large B-cell lymphoma (DLBCL) poses a challenge in the clinical practice. Overall, 35%–40% of DLBCL patients are primary refractory or relapse following a first-line therapy.^1^ In recent years, chimeric antigen receptor (CAR)-T cell therapies targeting malignant lymphocytes by genetically modified autologous T cells expressing CD19-targeting CARs were introduced in the clinical practice.^2,3^ Currently, 3 CAR-T cell products are FDA licensed for r/r DLBCL: axi-cel (axicabtagene ciloleucel), liso-cel (lisocabtagene maraleucel), and tisa-cel (tisagenlecleucel).^4–7^ Treatment with axi-cel and liso-cel has achieved long-term remissions in about 40% of primary-refractory/early relapsed DLBCL patients following first-line therapy and both are approved in these settings.^5,7^ Tisa-cel is available for DLBCL patients relapsing after 2 preceding therapy lines.^6^

While an extensive expertise has been gathered in the field of CAR-T cell therapy and B-cell lymphoproliferative malignancies with systemic manifestations, still little is known regarding efficacy and side effects of CAR-T cell therapy in those patients presenting with central nervous system (CNS) disease.^8–11^

r/r DLBCL patients with CNS involvement represent an unmet clinical need. Commonly, high-dose methotrexate (MTX)-based and/or cytarabine (ARA-C)-based polychemotherapy penetrating the blood-brain barrier is administered. However, this therapy is accompanied by substantial side effects such as cytopenia, organ injuries (eg, kidney, liver, lung, mucosa), and leukoencephalopathy and efficacy is limited.^12^ Whole-brain radiotherapy represents another treatment option in this patient collective but is associated with relevant neurotoxicity occurring over a time course of months to years. By means of conventional immunochemotherapy, the outcomes remain poor with the median overall survival (OS) in secondary CNS lymphoma (SCNSL) accounting for 3.9 months only.^13^ Acknowledging the efficacy of CAR-T cell therapy in r/r patients with non-CNS DLBCL, the question arises whether this approach can be safely and effectively applied in r/r patients with CNS manifestations. Initially, patients with CNS manifestations were excluded from all pivotal CAR-T cell studies taking into consideration concerns about immune effector-cell–associated neurotoxicity syndrome (ICANS) and the capability of CAR-T cells to cross the CNS barrier. Thus, only few data exist regarding outcomes and toxicities of CAR-T cell therapy in patients with CNS manifestation. Within a recent phase 1/2 clinical trial with 12 relapsed patients with primary CNS lymphoma (PCNSL), tisa-cel resulted in a complete response (CR) rate of 50% being well-tolerated with no treatment-related deaths.^14^ In the previous study with liso-cel, 4 of 9 patients (44%) with SCNSL achieved an objective response post-CAR-T with low incidence of cytokine release syndrome (CRS) (1/9) and neurological events (1/9).^15^ Similarly, Ahmed et al. reported a CR rate of 85.7% among 7 patients with SCNSL who underwent CAR-T cell therapy. No grade 4 ICANS was reported in this subset of these patients.^16^ Along this line, CAR-T cells were shown to be able to cross the CNS barrier, as well as to expand and persist in CNS compartments.^14,17,18^

Aiming to further reveal the efficacy and toxicity of CAR-T cells in r/r DLBCL patients with CNS manifestation in a real-world setting, we analyzed all cases with CNS disease treated with CAR-T cells in our academic center. Furthermore, we compared the outcomes in CNS group with those receiving CAR-T cell therapy due to non-CNS B-cell malignancy.

## METHODS

### Patient cohorts and study design

This retrospective study enrolled 80 consecutive r/r DLBCL patients with (n = 15) and without (n = 65) CNS manifestations treated with CAR-T cell therapy between January 2019 and August 2022 at the University Hospital/Inselspital, Bern, Switzerland. Both CAR-T cell products applied within this period in our center were considered: axi-cel and tisa-cel. Clinical data were gathered from the medical records, electronic patient files, and electronic database and supplemented by additional patient-related documents. The patients included in the analysis were divided into 2 cohorts for comparative analysis: with and without CNS involvement at the time point of CAR-T cell application. The study was approved by the local ethics committee (Kantonale Ethikkommission Bern, decision number 2022-00203 from May 4, 2022), and all patients signed informed consent and/or did not declare refusal to participate. All study procedures were performed in accordance with relevant guidelines, such as the Declaration of Helsinki, as well as local regulations.

### Patient stratification, response assessment, infectious prophylaxis, and adverse event grading

Previous to CAR-T cell administration, all patients received lymphodepleting chemotherapy with fludarabine (30 mg/m^2^ IV per day on days −5 to −3) and cyclophosphamide (300 mg/m^2^ IV on day −5 for tisa-cel; 500 mg/m^2^ IV on day −5 for axi-cel). CAR-T cell infusion was performed on day 0. Grading of CRS and ICANS was performed according to the American Society for Transplantation and Cellular Therapy consensus grading for CRS and ICANS.^19^ Response to CAR-T cell therapy was classified as CR, partial response (PR), stable disease, and progressive disease (PD). Overall response rate (ORR) was defined as the proportion of patients who achieved CR or PR, and complete response rate (CRR) as the proportion of patients with CR. CR for patients with synchronous (CNS and non-CNS) disease required CR for both CNS and non-CNS lesions. Response was assessed using common radiological criteria based on computer topographies and/or MRI performed 1 month after CAR-T cell infusion, as well as MRI and/or positron emission tomography and computed tomography performed 3, 6, and 12 months after CAR-T cell infusion.

Standard infectious prophylaxis was initiated irrespective of CNS disease status at the time of CAR-T cell therapy. Aciclovir (2 × 400 mg per day) and cotrimoxazole (2 × 960 mg twice a week or 3 × 960 mg per week) were mandatory for all CAR-T cell patients. Immunoglobulin replacement therapy was recommended for patients with serum IgG levels below 4 g/L and recurrent or severe infections. Primary prophylaxis with granulocyte colony-stimulating factors was administered if the absolute neutrophil count was decreasing below 1000/mm^3^. The use of antiepileptics was not used as a primary prophylaxis and was initiated only in symptomatic patients with CNS manifestations. The toxicity management guidelines were uniform irrespective of CNS manifestations. Particularly, there were no differences in management of CRS and/or ICANS such as timing of intervention with steroids and/or tocilizumab. Adverse events post-CAR-T cell therapy were assessed according to the recommendations of Common Terminology Criteria for Adverse Events (CTCAE), Version 5.0.^20^ Data cutoff for evaluation of outcomes was March 31, 2023.

### Endpoints and statistical analysis

The primary end points of this study were ORR, progression-free survival (PFS), and OS in the CNS versus non-CNS cohorts. Secondary endpoints were incidence and severity of CRS and ICANS following CAR-T cell therapy in the CNS versus non-CNS cohorts.

For categorical data, Fisher exact test was used. The unpaired *t* test was applied for normally distributed metrical data. In case of not normally distributed metrical data, the Mann-Whitney *U* test was used. PFS and OS were analyzed by Kaplan-Meier method. For PFS calculations, events were defined as disease progression or death, whereas for OS death only was considered for event definition. *P* values less than 0.05 were considered significant. Descriptive statistics, Kaplan-Meier curves, the calculation of *P* values, and the creation of figures were conducted with GraphPad Prism 9.0.1 for Windows (GraphPad Software, San Diego, California, USA). Multivariable analysis was performed with R version 4.1.2.

## RESULTS

### Patient baseline characteristics

Patient baseline characteristics are summarized in Table 1 for all patients and in detail in Suppl. Table S1 for those with CNS lymphoma. In total, 80 patients were included in the study. Of them, 15 (19%) had CNS (termed CNS group) and 65 (81%) had non-CNS manifestations only (non-CNS group). The frequency of secondary CNS manifestations (11/15, 73%) was significantly higher than primary CNS manifestations (4/15, 27%; 3 PCNSL, 1 DLBCL with synchronic peripheral and CNS manifestations) (*P* < 0.001). Parenchymal involvement was the most common CNS manifestation (15/15, 100%) followed by leptomeningeal (6/15, 40%) and central nerve lesions (3/15, 20%). The median time from diagnosis to CAR-T cell therapy was numerically shorter in the CNS group: 12 versus 24 months, *P* = 0.103. Otherwise, both cohorts were comparable regarding baseline clinical characteristics (gender, age, proportion of primary refractory patients, and those being refractory to the last treatment, Eastern Cooperative Oncology Group (ECOG) at CAR-T cell therapy) (Table 1). The median number of therapy lines before CAR-T cell treatment was 3 in both CNS and non-CNS groups (*P* = 0.631). In total, 6 of 15 patients (40%) from CNS group underwent MTX-containing prophylaxis of CNS relapse within first-line treatment: 4 with conventional triple intrathecal therapy (methotrexate/cytarabine/prednisone) and 2 with alternating intravenous MTX (Suppl. Table S1). Median time from indication of CAR-T cell therapy to treatment administration was similar among patients with CNS and non-CNS manifestations: 2.6 versus 2.5 months (*P* = 0.345).

**Table 1.** Characteristics of Patients Preceding CAR-T Cell Therapy

Parameters	Pts with CNS Manifestation	Pts without CNS Manifestation	*P* Value	All Patients
**Number of patients (%**)	15 (19%)	65 (81%)	-	80 (100%)
**Age (range**)	61 (20–79)	68 (18–82)	0.670	63 (36–79)
**Gender, n (%**)				
Male	8 (53%)	41 (63%)	0.513	49 (61%)
Female	7 (47%)	24 (37%)		31 (39%)
**Diagnosis**				
DLBCL in total, n (%)	15 (100%)	63 (97%)	0.057	78 (98%)
-De novo	13 (87%)	36 (55%)	0.056	49 (61%)
-PCNSL, n (%)	3 (20%)	0 (0%)	0.006	2 (3%)
-PMBCL, n (%)	0 (0%)	2 (3%)	1.000	2 (3%)
*-Secondary*	2 (13%)	28 (43%)	0.039	30 (38%)
-Transformed FL	2 (17%)	18 (28%)	0.333	20 (25%)
-Transformed MZL	0 (0%)	3 (5%)	1.000	3 (4%)
-Transformed B-CLL/SLL	0 (0%)	5 (8%)	0.578	5 (6%)
FL, n (%)	0 (0%)	2 (3%)	1.000	2 (3%)
**Description of CNS manifestation**				
Primary CNS manifestation at diagnosis	4 (27%)	-	<0.001	-
Secondary CNS manifestation at relapse/progress	15 (100%)	-	-	-
**CNS sites affected**				
Parenchymal lesions, n (%)	15 (100%)	-	-	-
Nerve lesions, n (%)	3 (20%)	-	-	-
Leptomeningeal, n (%)	6 (40%)	-	-	-
**Median number of therapy lines before CAR-T, n (range**)	3 (2–5)	3 (2–7)	0.631	3 (2–7)
**Prior autologous SCT, n (%**)	9 (60%)	30 (46%)	0.398	39 (49%)
**Prior allogeneic SCT, n (%**)	0 (0%)	0 (0%)	-	0 (0%)
**Primary refractory, n (%**)	9 (60%)	22 (34%)	0.080	31 (48%)
**Refractory to last treatment, n (%**)	9 (60%)	46 (71%)	0.538	55 (69%)
**Median time from diagnosis to CAR-T, months (range**)	12 (4–69)	24 (4–233)	0.103	19 (4–233)
**ECOG at CAR-T cell therapy (77/80 available**)				
ECOG 0–2, n (%)	13 (87%)	57 (71%)	0.796	70 (88%)
ECOG >2, n (%)	1 (7%)	6 (9%)	-	7 (9 %)
**Time from CAR-T-indication to CAR-T cell therapy itself**	2.6 (1–5)	2.5 (1–9)	0.345	3 (1–9)

B-CLL/SLL = B-cell lymphocytic leukemia/small lymphocytic lymphoma; CAR-T = chimeric antigen receptor T cells; CNS = central nervous system; DLBCL = diffuse large B-cell lymphoma; ECOG = Eastern Cooperative Oncology Group; FL = follicular lymphoma; IPI = International Prognostic Index; MZL = marginal zone lymphoma; PCNSL = primary central nervous system lymphoma; PMBCL = primary mediastinal large B-cell lymphoma; Pts = patients; SCT = stem cell transplantation.

### Disease features and CAR-T cell treatment

Disease features and characteristics of CAR-T cell therapy are presented in Table 2. Disease status before CAR-T cell therapy was most frequently PD and PR in both groups: 60% and 40% in the CNS group, and 45% and 42% in the non-CNS group, respectively (*P* > 0.05).

**Table 2.** Disease Features and Characteristics of CAR-T Cell Therapy and Outcomes Among the Patients of the Study

Parameters	Pts with CNS Manifestation	Pts without CNS Manifestation	*P* Value	All Patients
**Remission at CAR-T cell therapy, n (%**)				
CR	0 (0%)	5 (8%)	0.580	5 (6%)
PR	6 (40%)	27 (42%)	0.772	33 (41%)
SD	0 (0%)	8 (12%)	0.347	8 (10%)
PD	9 (60%)	29 (45%)	0.578	38 (48%)
**CAR-T cell product, n (%**)				
All products	15 (100%)	65 (100%)	0.763	80 (100%)
Axicabtagene ciloleucel (axi-cel)	4 (27%)	21 (32%)	0.764	25 (31%)
Tisagenlecleucel (tisa-cel)	11 (73%)	44 (68%)	1.000	55 (69%)
**Cyclophosphamide/fludarabine lymphodepletion**	15 (100%)	65 (100%)	-	80 (100%)
**CRS after CAR-T cell therapy, n (%**)				
Grade 0	1 (7%)	13 (20%)	0.450	14 (18%)
Grade 1	9 (60%)	30 (46%)	0.400	39 (49%)
Grade 2	4 (27%)	21 (32%)	0.766	25 (31%)
Grade 3	1 (7%)	1 (2%)	0.342	2 (3%)
Grade 4	0 (0%)	0 (0%)	-	0 (0%)
**ICANS after CAR-T cell therapy, n (%**)				
Grade 0	7 (47%)	46 (71%)	0.128	53 (66%)
Grade 1	3 (20%)	4 (6%)	0.021	7 (9%)
Grade 2	2 (13%)	6 (9%)	0.640	8 (10%)
Grade 3	2 (13%)	6 (9%)	0.640	8 (10%)
Grade 4	0 (0%)	4 (6%)	0.700	4 (5%)
**Treatment of CRS and/or ICANS, n (%**)				
Steroids	11 (73%)	34 (52%)	0.161	45 (56%)
Tocilizumab	11 (73%)	42 (65%)	0.763	53 (66%)
**Median time to best response, months (range**)	1.1 (0.4–3.7)	2.7 (0.1–13.1)	<0.001	2.7 (0.1–13.1)
**Detection of best response, n (%**)				
CT	3 (20%)	19 (29%)	-	22 (28%)
MRI	11 (73%)	0 (0%)	-	8 (10%)
PET-CT	5 (33%)	44 (68%)	-	49 (61%)
CSF	1 (7%)	0 (0%)	-	1 (1%)
Bone marrow biopsy/MRD	0 (0%)	0 (0%)	-	0 (0%)
**Best response after CAR-T cell therapy, n (%**)				
Overall response rate (CR + PR), n (%)	12 (80%)	52 (80%)	1.000	64 (80%)
CR	3 (20%)	27 (42%)	0.197	30 (38%)
PR	9 (60%)	25 (38%)	0.310	34 (43%)
SD	1 (7%)	0 (0%)	0.215	1 (1%)
PD	2 (13%)	10 (15%)	1.000	12 (15%)
Not available	0 (0%)	2 (3%)	-	2 (3%)
**r/r disease following CAR-T cell therapy, n (%**)	6 (40%)	27 (42%)	1.000	33 (41%)
CNS lesions	3 (20%)	1 (2%)	0.020	4 (5%)
Non-CNS lesions	2 (13%)	26 (40%)	0.071	28 (35%)
CNS + non-CNS lesions	1 (7%)	0 (0%)	0.188	1 (1%)
**Median time to r/r disease following CAR-T cell therapy, months (range**)	2.6 (1–9.1)	3.1 (0.4–27.3)	0.641	3.1 (0.4–27.3)
**Median time follow-up, months (range**)	4.9 (1–39.5)	11.1 (0.1–44.9)	<0.001	8.7 (0.1–44.9)
**Remission status at last follow-up, n (%**)				
CR	4 (27%)	32 (50%)	0.310	36 (45%)
PR	5 (33%)	12 (18%)	0.426	17 (21%)
SD	1 (7%)	0 (0%)	0.342	1 (1%)
r/r disease	6 (40%)	27 (42%)	1.000	33 (41%)
Not available	0 (0%)	1 (2%)	-	1 (1%)
**Survival status at last follow-up, n (%**)				
Alive	7 (47%)	33 (51%)	1.000	40 (50%)
Dead	8 (53%)	32 (49%)		40 (50%)
**Mortality reasons, n (%**)				
r/r lymphoma	3 (20%)	16 (25%)	0.430	19 (24%)
Not available	3 (20%)	4 (6%)		7 (9%)
Nonlymphoma reasons	2 (13%)	12 (18%)		14 (18%)
-Infection	1 (7%)	8 (12%)		9 (11%)
-CRS/ICANS	0 (0%)	0 (0%)		0 (0%)
-Thromboembolism	0 (0%)	2 (3%)		2 (3%)
-Other reasons	1 (7%)	2 (3%)		3 (4%)

CAR-T = chimeric antigen receptor T cells; CNS = central nervous system; CSF = cerebrospinal fluid; CR = complete remission; CRS = cytokine release syndrome; CT = computed tomography; ICANS = immune effector-cell–associated neurotoxicity syndrome; MRD = minimal residual disease; PD = progressive disease; PET-CT = positron emission tomography and computed tomography; PR = partial remission; Pts = patients; r/r = relapsed/refractory; SD = stable disease.

In the CNS group, 11 (73%) patients received tisa-cel and 4 (27%) were treated with axi-cel. In the non-CNS group, tisa-cel was the most common CAR-T cell product used (44/65, 68%) followed by axi-cel (21/65, 32%). Before CAR-T, all patients from the non-CNS group underwent a bridging therapy presented by immunochemotherapy, immunochemotherapy in combination with radiotherapy, or radiotherapy only. Of the patients from CNS group, 12 patients (80%) underwent any kind of bridging therapy following indication to CAR-T cell therapy: systemic treatment with MTX-containing regimen (42%; 5/12) followed by ibrutinib and steroids (17%; 2/12 for each) as well as radiotherapy (8%; 1/12). Two remaining patients (17%) received systemic immunochemotherapy for non-CNS lesions following indication to CAR-T treatment but experienced PD with new CNS lesions preceding CAR-T cell administration (Suppl. Table S1). Best objective response to the bridging therapy was PR and documented in half of the patients (50%), while the remaining patients (50%; 6/12%) failed to respond and entered CAR-T cell therapy with PD.

### Feasibility and adverse events of CAR-T cell therapy in patients with CNS manifestations within first 100 days post-CAR-T

#### CRS and ICANS

**Figure 1.**
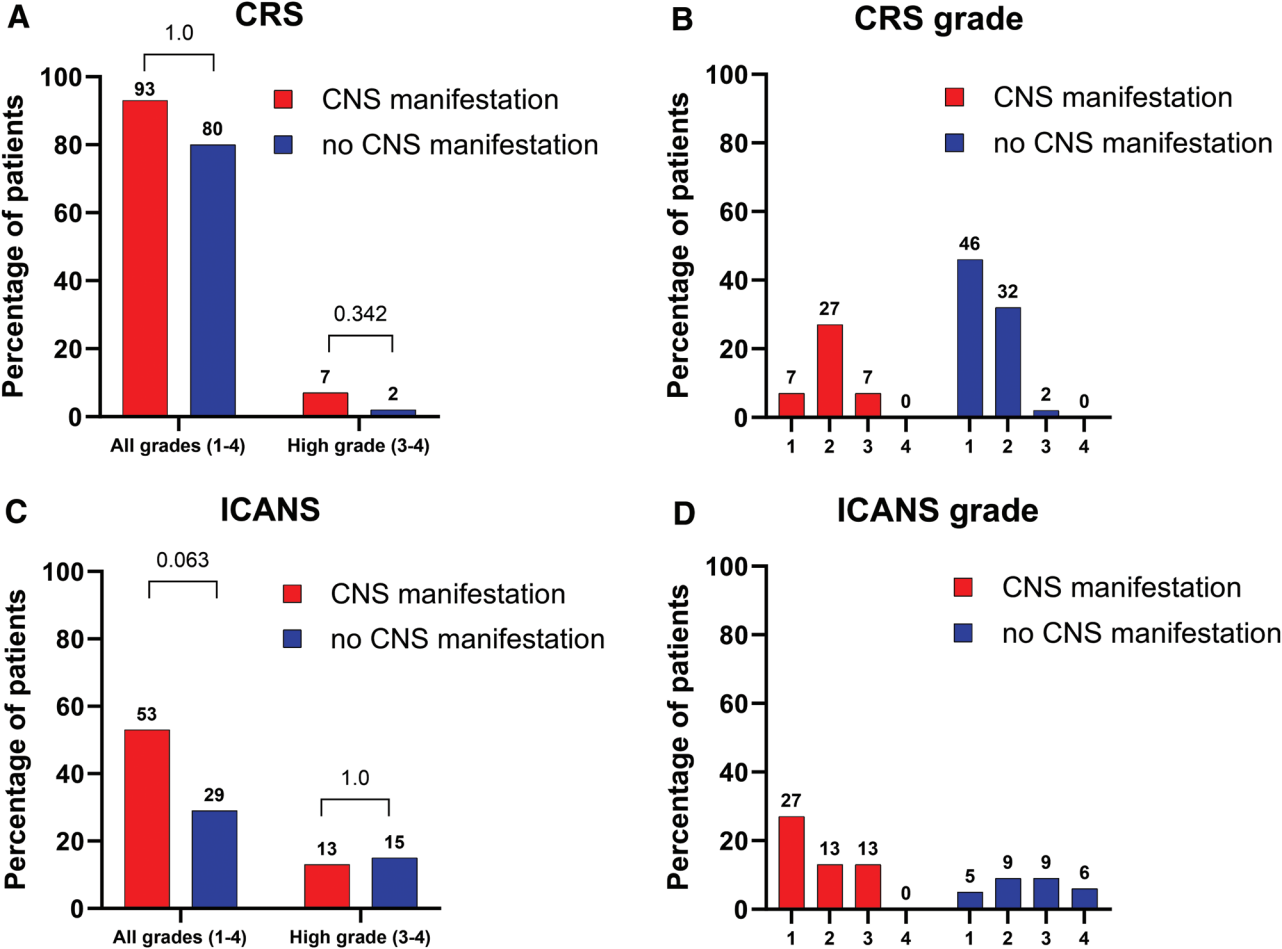
**Comparison of adverse events from CAR-T cell therapy in cohorts CNS vs non-CNS.** (A) Comparison of CRS incidence. (B) Visualization of CRS grade distribution. (C) Comparison of ICANS incidence. (D) Visualization of ICANS grade distribution. CAR-T = chimeric antigen receptor T cells; CNS = central nervous system; CRS = cytokine release syndrome; ICANS = immune effector-cell–associated neurotoxicity syndrome.

The frequency of CRS, ICANS and their severity are presented in Table 2, Suppl. Table S2, and Figure 1. The frequency of all CRS grades (grades 1–4) was comparable in the CNS and non-CNS groups: 93% versus 80%; *P* = 1.0 (Figure 1A). Grade 3 CRS was documented in 1 case in each cohort (7% CNS versus 2% non-CNS, *P* = 0.342), while grade 4 CRS did not occur (Figure 1B). There were no differences in CRS frequency and grading between both CAR-T cell products (axi-cel and tisa-cel) in the CNS group (Suppl. Table S2).

Numerically, ICANS (all grades) was more frequent in patients with CNS lymphoma manifestation, although the difference was not statistically significant (53% versus 29%; *P* = 0.063) (Figure 1C). Similarly, grade 3 ICANS was numerically slightly more frequent in the CNS group (13% versus 9%, *P* = 0.640). Yet, no grade 4 events were documented in the CNS group, while 6% (4/65) of patients in the non-CNS group experienced grade 4 ICANS (Figure 1D; *P* = 0.700). Again, the frequency and grading of ICANS in the CNS group did not depend on applied CAR-T cell product (axi-cel or tisa-cel) in the CNS group (Suppl. Table S2).

Application of corticosteroids was more frequently documented in patients with CNS manifestation (73% versus 52%; *P* = 0.161). Tocilizumab was applied in 73% (11/15) and 65% (42/65) of patients within the CNS and non-CNS cohorts, respectively (*P* = 0.763). No deaths occurred related to CRS or ICANS.

### Feasibility and adverse events of CAR-T cell therapy beyond CRS/ICANS

The feasibility and adverse effects of CAR-T cell therapy beyond CRS/ICANS in patients with r/r DLBCL and CNS manifestations and who did not experience PD within first 100 days post-CAR-T (11/15) are presented in Suppl. Table S3. Three of 11 patients died due to nonlymphoma reasons: hemophagocytic lymphohistiocytosis, intestinal perforation, and infection, respectively. Of the remaining cases (8/11), only 1 patient (9%) presented with worse ECOG performance status (0→1) following CAR-T cell therapy. Following hematologic toxicities were observed: worsening of anemia in 36% of patients (4/11; grades 2, 3, and 4), of thrombocytopenia in 64% (7/11; grade 1, 2× grade 2, 1× grade 3, and 3× grade 4), and of leukopenia in 27% (3/11, 2× grade 2, 1× grade 3). The most common nonhematologic toxicity was infection documented in 9 of 11 patients with available data (82%): grade 1 in 9% (1/11), grade 2 in 36% (4/11), grade 3 in 27% (3/11), and grade 5 in 18% (2/11). One of the patients with grade 5 infection experienced intestinal perforation leading to peritoneal infection and death subsequently.

### Treatment outcomes

**Figure 2.**
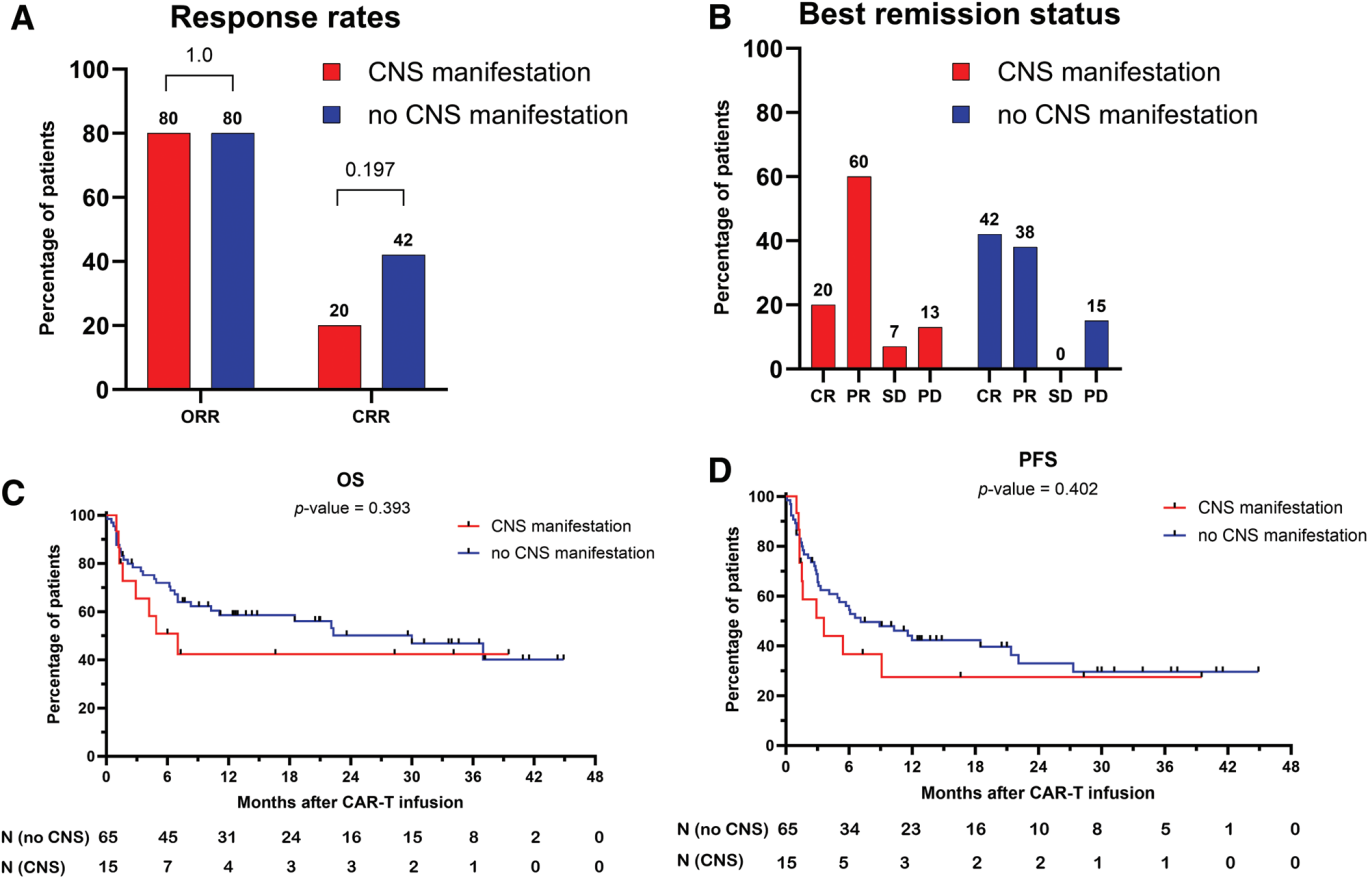
**Comparison of outcomes of CAR-T cell therapy in cohorts CNS vs non-CNS.** (A) Comparison of ORRs and CR rates. (B) Distribution of best remission status. (C) OS. (D) Progression-free survival (PFS). CAR-T = chimeric antigen receptor T cells; CRR = complete response rate; CNS = central nervous system; ORR = overall response rate; OS = overall survival; PD = progressive disease; PR = partial remission; SD = stable disease.

**Figure 3.**
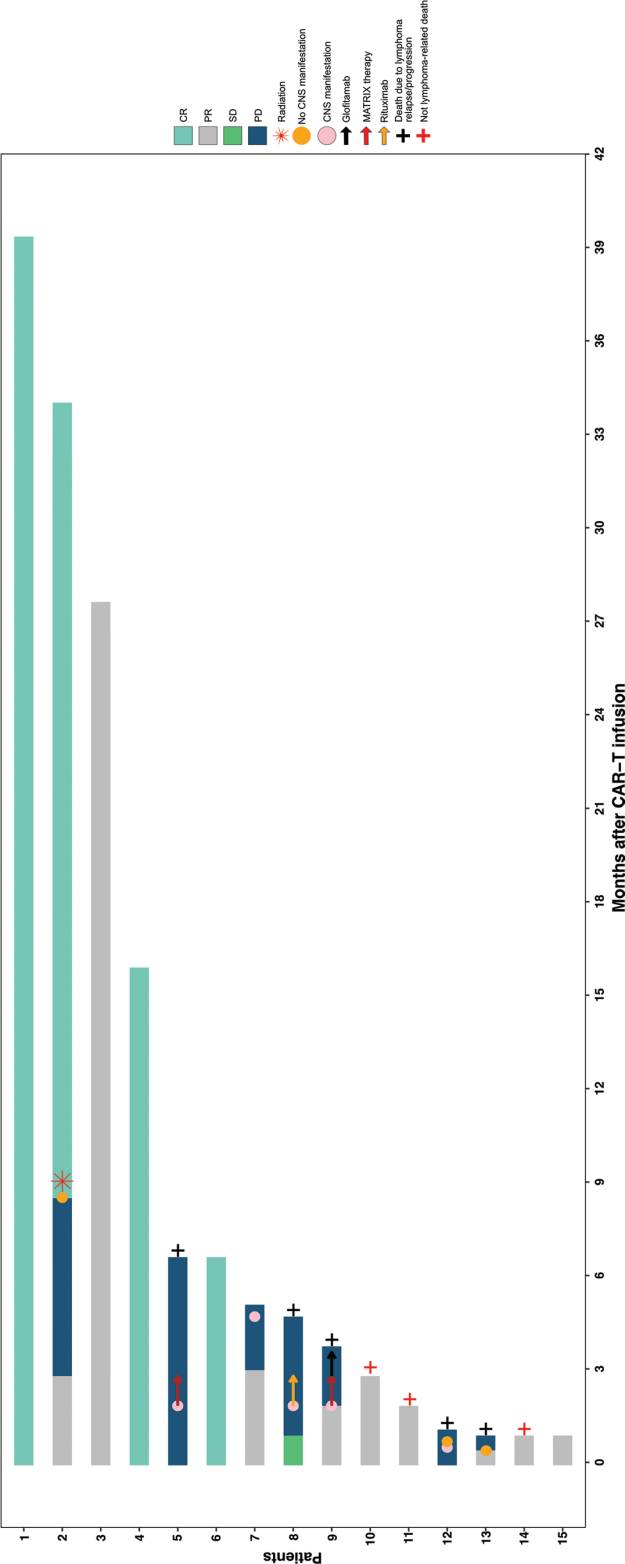
**Post-CAR-T outcomes and subsequent lymphoma therapies in patients with CNS manifestations preceding CAR-T cell therapy.** CAR-T = chimeric antigen receptor T cells; CNS = central nervous system; CR = complete remission; MATRIX = methotrexate, cytarabine, thiotepa, rituximab; PD = progressive disease; PR = partial remission; SD = stable disease.

The outcomes of CAR-T cell therapy are presented in Table 2, Suppl. Tables S1-S2, and Figures 2 and 3. Best responses following CAR-T cell therapy were achieved with medians of 1.1 and 2.7 months in CNS and non-CNS groups, respectively (*P* < 0.001). ORRs (80% versus 80%; *P* = 1.0) and CRRs (20% versus 42%; *P* 0.197) were comparable for both cohorts (Figure 2A and 2B). Within the CNS group, the type of CAR-T cell product (axi-cel or tisa-cel) did not impact response and survival outcomes (Suppl. Table S2). No significant differences were observed for estimated PFS (*P* = 0.157) and OS (*P* = 0.393). In the CNS group, median PFS (mPFS) and median OS (mOS) were 3.6 months (95% CI [confidence interval], 1.5-NR; *P* = 0.2) and 7.0 months (95% CI, 2.9-NR; *P* = 0.400), and in the patient population without CNS manifestation, 11 (95% CI, 5.1-27.3) and 30 (95% CI, 10.3-NR) months, respectively (Figure 2C, 2D).

Median follow-up was shorter for the CNS cohort due to chronologically later treatment dates (4.9 versus 11.1 months; *P* < 0.001). In total, 40% (6/15) and 42% (27/65) CNS and non-CNS patients were documented with a relapse/progressive disease in both groups (*P* = 1.000) with a median time to relapse of 2.6 and 3.1 months, accordingly (*P* = 0.641). Four of 7 patients (57%) from the CNS group had CNS relapse/progression (#5, 7, 8, 9), while 3 other patients (43%) developed relapse either beyond CNS (2/6; #2, 13) or presented with both, CNS and non-CNS lymphoma manifestations, at progression (1/6; #12; Figure 3). In the non-CNS cohort, only 1 of 27 patients (4%) showed CNS involvement at relapse. Four of the patients from the CNS group and who experienced relapse/progression post-CAR-T (4/7) underwent subsequent lymphoma therapy: MATRIX (2/4; #5, 9), radiotherapy (1/4; #2), and rituximab only (1/4; #8) (Figure 3). One patient received glofitamab (#9) following progression beyond CNS and post-MATRIX therapy. Of these 4 cases, only 1 patient (#2) could regain CR after local radiotherapy of solitary pulmonary lymphoma lesion. The remaining patients succumbed to PD. Note: # symbolizes the exact number of patients.

Fifty-three percent (8/15) and 49% (32/65) patients died in CNS and non-CNS cohorts (*P* = 1.000). Mortality was mostly due to disease progression: 20% (3/15) in the CNS and 25% (16/65) in the non-CNS groups (*P* = 1.000). Infection was the most common event among nonlymphoma death reasons: 7% (1/15) in CNS and 12% (8/65) in non-CNS groups, accordingly. No patients succumbed to CRS or ICANS.

## DISCUSSION

While more extensive data regarding efficacy and toxicity of CAR-T cell therapy have been generated for patients with r/r DLBCL without CNS involvement, the role of CAR-T cells in patients with CNS involvement remains to be further investigated. In our study, performed at a single tertiary academic center, we focused on the outcomes and safety of CAR-T cell therapy in DLBCL patients with CNS versus without CNS involvement undergoing CAR-T cell treatment between 2019 and 2022.

Eighty patients have been included in our retrospective study: 15 (19%) with CNS and 65 (81%) without CNS involvement. Despite size difference, both cohorts were balanced as per basal patient and disease characteristics.

Interestingly, no differences in best response were documented between the CNS and non-CNS groups, while numerically patients with peripheral disease had a higher CR rate than those with CNS manifestation (42% versus 20%, respectively; *P* = 0.197). Furthermore, both groups had almost the same relapse/progression rate (40% CNS versus 42% non-CRS; *P* = 1.000) and mortality rate (53% CNS versus 49% non-CNS; *P* = 0.1000). Accordingly, although mPFS was numerically better in the non-CNS group, no statistically significant differences in mPFS and mOS were observed between both groups. For the CNS group, mPFS was 3.6 months and mOS 7.0 months, whereas the mPFS and mOS were 11 and 30 months in the non-CNS group, respectively (for PFS, *P* = 0.157; for OS, *P* = 0.393).

Considering response rates, our data were in agreement with previous studies reporting on the efficacy of CAR-T cell therapy in r/r DLBCL patients with CNS manifestations.^14,21^ In a recent meta-analysis of all published data describing CAR-T cell use in r/r CNS lymphoma, Cook et al. identified 128 patients with PCNSL (30/128) and SCNSL (98/128). 56% PCNSL and 47% SCNSL patients achieved a CR with 37% of patients remaining in remission at 6 months in both groups.^21^ Although the rate of CR was lower in our CNS cohort (28%), 50% of patients remained in remission at 6 months. By analogy to our analysis, Bennani et al. analyzed the outcomes of 17 lymphoma patients who had a history of secondary CNS involvement or had active CNS disease at the time of CAR-T cell infusion with axi-cel. The results were compared with 283 lymphoma patients with non-CNS manifestation who also underwent axi-cel. All leukapheresis patients were included in the intention-to-treat analysis for response rate and event-free survival (EFS). The CAR-T infusion rate was 88% for the CNS cohort (15/17) compared to 93% (262/283) in the non-CNS cohort. With a median follow-up of 10.1 months from leukapheresis (range 7.6–12.6), the intention-to-treat best ORRs (CR + PR) and ongoing responses at month 6 between CNS and non-CNS cohorts were 75% versus 59%, and 41% versus 31%, respectively. EFS from leukapheresis was not statistically significantly different between CNS and non-CNS cohorts (6 month EFS: CNS cohort, 36%; non-CNS cohort, 57%; HR = 1.58, 95% CI, 0.83-3.01, *P* = 0.16). Six month EFS from the date of infusion for the CNS cohort was 49.9%.^22^ However, longer-term response persistence remains to be investigated. Overall, our and previous findings demonstrate encouraging response rates to CAR-T cell therapy among patients with B-cell malignancies and CNS disease.

The frequency of all CRS grades was similar in the CNS and non-CNS groups. Regarding neurotoxicity, ICANS of all grades showed a higher frequency in the CNS group without statistical significance (53% versus 29%; *P* = 0.063). Yet, the frequency of higher-grade^3,4^ ICANS (13% versus 15%; *P* = 1.0) was similar in both groups.

In a meta-analysis of 128 PCNSL and SCNSL patients by Cook et al., ICANS occurred in roughly half of each cohort, with 18% and 26% documented grade 3 and 4 neurotoxicity.^21^ These data were in accordance with our findings and comparable to those reported in the ZUMA-1 (G3/4 ICANS: 28%), ZUMA-2 (G3/4 ICANS: 21%), and JULIET (G3/4 ICANS: 12%) studies.^4,6,23^ Again, Bennani et al. reported on the comparable incidence of CRS and ICANS, of any grade or grade 3 or higher, between the CNS and non-CNS cohorts after axi-cel infusion.^22^ Thus, CNS disease does not appear to be associated with more severe neurotoxicity and should not prevent patients from receiving CAR-T cells.

To date, few data are available regarding the feasibility and safety of CAR-T cell therapy in patients with CNS manifestations beyond CRS and ICANS. In our analysis, nonrelapse mortality (NRM) in the CNS group was 13% following CAR-T cell therapy. To date, there is no literature reporting on NRM in patients with CNS manifestations undergoing CAR-T cell therapy. In 2 large real-world studies addressing efficacy and safety of CAR-T cell therapy in r/r large B-cell lymphoma without CNS manifestations, the NRM was 4.4% and 6%, respectively.^24,25^ Although our study encompassed only 15 cases, the potential risk of increased NRM in this patient collective should be considered and patients should be selected carefully before CAR-T cell therapy. Particularly, our data underline the crucial relevance of prophylaxis and optimal management of infectious complications post-CAR-T cell therapy.^26^ Globally, 82% of patients in our study experienced infections, with 27% presenting with infection of at least grade 3. In contrast, observed hematologic toxicity following CAR-T cell therapy was mild and well manageable.

Limitations of our study are the relatively small number of patients with CNS involvement, the heterogeneity of patient collective as well as the retrospective and monocentric study design. Additionally, longer-term follow-up is required to provide further insights into post-CAR-T cell therapy outcomes in patients with CNS disease.

## CONCLUSIONS

Results from our study and other analysis quote a support in terms of application of CAR-T cells in DLBCL patients with primary and secondary CNS. Additionally, our study underlies the fact that enhanced vigilance is required for prophylaxis and thorough management of infectious complications in these patients. To sum up, CAR-T cell therapy should not be withheld for DLBCL patients with CNS manifestations.

## ACKNOWLEDGMENTS

The authors wish to thank the nursing and physician staff at the University Hospital Bern and its associated partner hospitals and collaborators for documentation of data relevant for this study. The authors also thank the patients and their families.

## AUTHOR CONTRIBUTIONS

The work reported in the article has been performed by the authors, unless clearly specified in the text. Conception and design: ES, HB, UB, TP. Data curation: ES, HB, PM, UB, TP. Analysis and interpretation of data: ES, HB, PM, UB, TP. Investigation: ES, HB, PM. Writing—original draft: ES, HB, UB. Writing—article review & editing: DA, MD, UN, GL, UB, TP. Resources: HB, PM, MD, UN. Supervision: UB and TP. All authors have read and approved the final article.

## DATA AVAILABILITY STATEMENT

The data sets used and/or analyzed during the current study are available from the corresponding author on reasonable request.

## DISCLOSURES

ES received honoraria from Amgen, BMS, Stemline, Sanofi, Incyte, Janssen, Takeda and JAZZ. GL received research grants not related to this article from AGIOS, AQUINOX, AstraZeneca, Bayer, Celgene, Gilead, Janssen, Morphosys, Novartis, Roche, and Verastem. GL received honoraria from ADC Therapeutics, Abbvie, Amgen, AstraZeneca, Bayer, BMS, Celgene, Constellation, Genase, Genmab, Gilead, Hexal-Sandoz, Immagene, Incyte, Janssen, Karyopharm, Lilly, Miltenyi, Morphosys, NanoString, Novartis, PentixaPharm, Roche, Sobi, and Takeda. All the other authors have no conflicts of interest to disclose.

## SOURCES OF FUNDING

The authors declare no sources of funding.

## Supplementary Material


