# Supplementary material for: CAR-T Cell Therapy Shows Similar Efficacy and Toxicity in Patients With DLBCL Regardless of CNS Involvement
Source: Hemasphere. 2023 Nov 30;7(12):e984. doi: 10.1097/HS9.0000000000000984 (PMC10691788; doi:10.1097/HS9.0000000000000984)
Supplement: Supplementary file 1 [file hs9-7-e984-s001.docx]

**Supplemental Table S1.** Treatment modalities and outcomes of DLBCL patients with CNS disease prior and after CAR-T cell therapy in details. CNS, central nervous system, CAR-T, chimeric antigen receptor; non-CNS, non-central nervous system; FU, follow-up; DLBCL, diffuse large B-cell lymphoma; R-CHOP, rituximab, cyclophosphamide, doxorubicin, vincristine; HDCT/ASCT, high-dose therapy and autologous stem cell transplantation; PD, progressive disease; MTX, methotrexate; i.v., intravenous therapy; 1L, first line therapy; MATRIX, methotrexate, rituximab, cytarabine, thiotepa; MRI, magnetic resonance imaging; CR, complete remission; PET-CT, positron emission tomography-computed tomography; PR, partial remission; Tisa-cel, tisagenlecleucel; R-DHAP, rituximab, dexamethasone, cytarabine, cisplatin; R-ICE, rituximab, ifosfamide, carboplatin, etoposide; RTX, radiotherapy; Gy, gray; HGBL, High-grade B-cell lymphoma; ARA-C, cytarabine; DA-EPOCH-R, dose-adjusted, etoposide, prednisone, vincristine, cyclophosphamide, doxorubicin, rituximab; R-DHAOx, rituximab, dexamethasone, oxaliplatin, cytarabine; Axi-cel, axicabtagene ciloleucel; PCNHL, Primary Cerebral Non-Hodgkin’s Lymphoma; Pola-BR, polatuzumab-bendamustine-rituximab; CT, computed tomography; FL, follicular lymphoma; progressive disease; HLH, haemophagocytosis; R-GemOx, rituximab, gemcitabine, oxaliplatin.

| **#patient/primary diagnosis** | **Therapies prior to indication to CAR-T cell therapy→ time point of CNS manifestation** | **CNS prophylaxis as part of initial treatment** | **Bridging therapy following indication to CAR-T cell therapy (# of cycles)** | **Best CNS response to bridging therapy/**  **remission status prior to CAR-T cells** | **Administered CAR-T cell product** | **Response assessment post-CAR-T therapy** | **Summary of best objective response (CNS+non-CNS lesions) to CAR-T cell therapy** | **Status at last FU** |
| --- | --- | --- | --- | --- | --- | --- | --- | --- |
| #1/DLBCL | 1.R-CHOP(x6) followed by front-line HDCT/ASCT→  PD with new CNS lesions | High-dose MTX i.v. (x3) within 1L | MATRIX (x3) | MRI (CNS lesions): CR  PET-CT (non-CNS lesions): PR | Tisa-cel | MRI (CNS lesions): CR  PET-CT (non-CNS lesions): CR | CR | Alive in CR 39 months post CAR-T |
| #2/DLBCL | 1. R-CHOP (x6)→ early relapse  2. R-DHAP (x1)→ PD  3. R-ICE (x1) followed by HDCT/ASCT→ PD with non-CNS and new CNS lesions | No | No | PD CNS and non-CNS | Tisa-cel | PET-CT (CNS and non-CNS lesions): CR; later PD (pul-monal) followed by RTX 23x2 Gy | CR | Alive in CR  34 months post CAR-T |
| #3/HGBL with *MYC* and *BCL-2* rearrangements | 1. DA-EPOCH-R (x6)→ PD  2. R-DHAOx(x2)→ PD for non-CNS lesions and new CNS lesions | MTX/ARA-C/  prednisone  Intrathecal (x4) within 1L | High-dose R-MTX i.v. | MRI (CNS lesions): PD CT (non-CNS lesions): PD | Axi-cel | MRI (CNS lesions): CR  PET-CT (non-CNS lesions): PR | PR | Alive in PR 28 months post CAR-T |
| #4 PCNHL | 1. MATRIX (x1) followed by R-ICE (x3)→ PR (MRI) with HDCT/ASCT  2. RTX CNS→ CR (MRI) followed by late relapse with neurolymphomatosis | No | No | CNS relapse | Tisa-cel | PET-CT (CNS and non-CNS lesions): CR | CR | Alive in CR  17 months post CAR-T |
| #5/DLBCL | 1. R-CHOP (x4)→ PD  2. R-ICE (x2)→ PD  3. RTX mediastinum with 36 Gy  4. Pola-BR (x2)→ PD of non-CNS lesions, new CNS lesions | No | RTX for CNS lesions (12x2 Gy) and gemcitabine for non-CNS lesions | CT (CNS and non-CNS lesions): PR | Tisa-cel | MRI (CNS lesions): PD  PET-CT (non-CNS lesions): PR | PD | Death from PD 7 months  post CAR-T |
| #6/DLBLC | 1. R-CHOP (x6) followed by RTX→ PD with new CNS lesions in MRI  2. MATRIX (x2)/R-ICE (x2) followed by HDCT/ASCT→  PD of non-CNS and CNS lesions | No | Steroids | MRI (CNS lesions): PD  CT (non-CNS lesions): PD | Tisa-cel | MRI (CNS lesions): CR  CT (non-CNS lesions): CR | CR | Alive in CR 7 months post CAR-T |
| #7/PCNHL | 1.MATRIX (x3) followed by HDCT/ASCT→ early relapse of CNS lesions | No | Steroids | MRI (CNS lesions): PD;  no non-CNS lesions | Axi-cel | MRI (CNS lesions): PR  cerebral biopsy with CR | CR | Alive in CR 6 months post CAR-T |
| #8/PCNHL | 1. R-MTX-ARA-C+thiotepa (x4) followed by HDCT/ASCT→ early relapse of CNS lesions  2. RTX 35,2 Gy→ early relapse of CNS lesions | No | Ibrutinib 420 mg/d | MRI (CNS lesions): PR;  no non-CNS lesions | Tisa-cel | MRI (CNS lesions): PR | PR | Death from PD 5 months post CAR-T |
| #9/transformed HGBL with *MYC* and *BCL-2* rearrangements from FL | 1. R-CHOP (x6)→ early relapse  2. R-ICE with HDCT/ASCT→PD with new CNS and non-CNS manifestations | MTX/ARA-C/  prednisone  intrathecal (x1) within 1L | No | MRI (CNS lesions): PD  CT (non-CNS lesions): PD | Tisa-cel | MRI (CNS lesions): PD  PET-CT (non-CNS lesions): CR | PD | Death from PD 4 months post-CAR-T |
| #10/DLBCL | 1. R-CHOP (x8), RTX for contralateral testis→ late relapse with CNS manifestation in MRI  2. MATRIX (x3) followed by HDCT/ASCT→ late CNS relapse | MTX/ARA-C/  prednisone  intrathecal (x9) within 1L | MATRIX (x1) followed by R-ICE (x2) | MRI (CNS lesions): PR  CT (non-CNS lesions): CR | Axi-cel | MRI (CNS lesions): PR  CT (non-CNS lesions): CR | PR | Death, not lymphoma-related (HLH) 3 months post CAR-T |
| #11/testicular DLBCL | 1. R-CHOP+2R (x6), RTX for contralateral testis→ early relapse with new CNS lesions in MRI  2. MATRIX (x2)→ PD of CNS lesions | No | Ibrutinib 420mg/d | MRI (CNS lesions): PR  CT (non-CNS lesions): CR | Axi-cel | MRI (CNS lesions): PR  CR (non-CNS lesions): CR | PR | Death, not lymphoma-related (diverticulitis followed by colon perforation and septic shock) 2 months post CAR-T |
| #12/DLBCL | 1. R-CHOP (x6)→ PD  2. R-DHAP (x2)/R-ICE (x1) followed by HDCT/ASCT-→ early relapse of non-CNS lesions | MTX (x3) i.v. within 1L | RTX femur (15x2 Gy) and R-GemOx (x1) for non-CNS lesions | MRI (CNS lesions): PD  CT (non-CNS lesions): PR | Tisa-cel | CT (non-CNS lesions): PD  autopsy (CNS+ non-CNS lesions): PD | PD | Death from PD  1 month post  CAR-T |
| #13/DLBCL | 1. R-CHOP (x6)→ late relapse of non-CNS lesions 2. R-GemOx (x6)→ early relapse with new CNS lesions; non-CNS lesions PR | No | MATRIX (x2) | MRI (CNS lesions): PD  CT (non-CNS lesions): PR | Tisa-cel | MRI (CNS lesions): PR | PD | Death from PD  1 month post CAR-T |
| #14/HGBL with *MYC* and *BCL-6* rearrangements | 1. R-CHOP (x2)→ PD with CNS manifestations in liqour  2.MATRIX (x2) + R-CHOP (x4)→ CR for CNS and PD for non-CNS lesions | MTX/ARA-C/  prednisone  intrathecal (x2) within 1L | R-GemOx (x1) | MRI (CNS lesions): PD  PET-CT (non-CNS lesions): PD | Tisa-cel | MRI (CNS lesions): PR  CT (non-CNS lesions): PR | PR | Death due to sepsis in PR 1 month post-CAR-T |
| #15 transformed DLBCL from FL | 1. R-CHOP (x6)→ PD  2. R-ICE (x2)→ PD with non-CNS and CNS lesions | No | MATRIX (x1) | MRI (CNS lesions): PR  CT (non-CNS lesions): PR | Tisa-cel | MRI (CNS lesions): PR  CT (non-CNS lesions): PR | PR | Alive in PR 1 month post  CAR-T |
