# Supplementary material for: CAR-T Cell Therapy Shows Similar Efficacy and Toxicity in Patients With DLBCL Regardless of CNS Involvement
Source: Hemasphere. 2023 Nov 30;7(12):e984. doi: 10.1097/HS9.0000000000000984 (PMC10691788; doi:10.1097/HS9.0000000000000984)
Supplement: Supplementary file 2 [file hs9-7-e984-s002.docx]

**Supplemental Table S2.** Outcomes of DLBCL patients with CNS disease treated with Tisa-cel and Axi-cel. DLBCL, diffuse large B-cell lymphoma; CNS, central nervous system; Tisa-cel, tisagenlecleucel; Axi-cel, axicabtagene ciloleucel; Pts., patients; CAR-T, chimeric antigen receptor-T cell therapy; CR, complete remission; PR, partial remission; SD, stable disease; PR, progressive disease; CRS, cytokine release syndrome; ICANS, immune-effector cell-associated neurotoxicity syndrome; r/r, relapsed or refractory.

| **Parameters** | **Pts with Tisa-cel** | **Pts with Axi-cel** | ***p*-value** |
| --- | --- | --- | --- |
| **Patients with CNS lymphoma treated with CAR-T cells** | 11 (73%) | 4 (27%) |  |
| **Remission status prior to CAR-T cell therapy, n (%)** | | | |
| CR | 0 (0%) | 0 (0%) | 0.264 |
| PR | 3 (27%) | 3 (75%) |  |
| PD | 8 (73%) | 1 (25%) |  |
| **CRS after CAR-T cell therapy, n (%)** | | | |
| Grade 0 | 0 (0%) | 1 (25%) | 0.596 |
| Grade 1 | 7 (64%) | 2 (50%) |  |
| Grade 2 | 3 (27%) | 1 (25%) |  |
| Grade 3 | 1 (9%) | 0 (0%) |  |
| Grade 4 | 0 (0%) | 0 (0%) |  |
| **ICANS after CAR-T cell therapy, n (%)** | | | |
| Grade 0 | 6 (55%) | 1 (25%) | 0.477 |
| Grade 1 | 2 (18%) | 2 (50%) |  |
| Grade 2 | 2 (18%) | 0(0%) |  |
| Grade 3 | 1 (9%) | 1 (25%) |  |
| Grade 4 | 0 (0%) | 0 (0%) |  |
| **Best** **response after CAR-T cell therapy** | | | |
| Overall response rate (CR+PR), n (%) | 8 (73%) | 4 (100%) | 0.516 |
| CR | 3 (27%) | 0 (0%) | 0.534 |
| PR | 5 (46%) | 4 (100%) |  |
| SD | 1 (9%) | 0 (0%) |  |
| PD | 2 (18%) | 0 (0%) |  |
| **r/r disease following CAR-T cell therapy, n (%)** | 5 (46%) | 1 (25%) | 0.604 |
| **Median time to r/r disease following CAR-T cell therapy, months (range)** | 1.5 (1.0-9.1) | 5.4 (-) | - |
| **Median time follow-up, months (range)** | 4.9 (1.0-39.5) | 4.5 (1.6-28.3) | 0.949 |
| **Remission status at last follow-up, n (%)** | | | |
| CR | 4 (36%) | 0 (0%) | 0.363 |
| PR | 3 (27%) | 2 (50%) |  |
| SD | 0 (0%) | 1 (25%) |  |
| r/r disease | 4 (37%) | 1 (25%) |  |
| **Survival status at last follow-up, n (%)** | | | |
| alive | 5 (45%) | 2 (50%) | 1.000 |
| dead | 6 (55%) | 2 (50%) |  |
| **Mortality reasons, n (%)** | | | |
| r/r lymphoma | 5 (83%) | 0 (0%) | 0.464 |
| non-lymphoma reasons | 1 (17%) | 2 (100%) |  |
| - infection | 1(17%) | 0 (0%) | - |
| - other reasons | 0 (0%) | 2 (100%) |  |
