# Supplementary material for: CAR-T Cell Therapy Shows Similar Efficacy and Toxicity in Patients With DLBCL Regardless of CNS Involvement
Source: Hemasphere. 2023 Nov 30;7(12):e984. doi: 10.1097/HS9.0000000000000984 (PMC10691788; doi:10.1097/HS9.0000000000000984)
Supplement: Supplementary file 3 [file hs9-7-e984-s003.docx]

**Supplemental Table S3.** Feasibility and adverse effects of CAR-T cell therapy in patients with relapsed/refractory DLBCL and CNS manifestations, who did not experience relapse/progressive disease within first 100 days post-CAR-T. CTCAE, Common Terminology Criteria of Adverse Events; CAR-T, chimeric antigen receptor T-cells; ECOG, Eastern Cooperative Oncology Group; CNS, central nervous system; PD, progressive disease; N/A, not available; PE, pulmonary embolism; VZV, Varicella-Zoster Virus

| **Parameters, CTCAE grading system, version 5.0** | **Prior to CAR-T cells** | **d+100 post-CAR-T cells** |
| --- | --- | --- |
| **Patient #1** | | |
| ECOG | 1 | 1 |
| Anemia | grade 2 | grade 1 |
| Thrombocytopenia | grade 1 | grade 1 |
| Leukocytopenia | 0 | 0 |
| Infections | Infectious enterocolitis, grade 2 | Proctitis, grade 2;  Peripheral nerve infection, grade 2 |
| Non-hematologic toxicity/disability | Pulmonary hypertension, grade 1; Pleural effusion, grade 1; Ascites, grade 1; Cushingoid, grade 2 | Dyspnea, grade 2;  Arthralgia, grade 2;  Fatigue, grade |
| **Patient #2** | | |
| ECOG | 1 | 1 |
| Anemia | grade 2 | grade 2 |
| Thrombocytopenia | 0 | grade 1 |
| Leukocytopenia | 0 | 0 |
| Infections | Pneumonia, grade 3;  Enterocolitis, grade 3 | Pneumonia, grade 2 |
| Non-hematologic toxicity/disability | Pleural effusion, grade 3;  Urinary retention, grade 2 | Upper gastrointestinal hemorrhage, grade 2 |
| **Patient #3** | | |
| ECOG | 0 | 0 |
| Anemia | grade 1 | grade 1 |
| Thrombocytopenia | 0 | 0 |
| Leukocytopenia | 0 | 0 |
| Infections | Urinary tract infection, grade 3 | Pneumonia, grade 2 |
| Non-hematologic toxicity/disability | Seizure, grade 2; Thromboembolic event, grade 4;  Hyperglycemia, grade 3;  Oral mucositis , grade 3 | 0 |
| **Patient #4** | | |
| ECOG | 1-2 | 1 |
| Anemia | grade 2 | grade 2 |
| Thrombocytopenia | grade 1 | grade 2 |
| Leukocytopenia | 0 | grade 2 |
| Infections | Pneumonia, grade 3;  Sepsis, grade 4 | no infection |
| Non-hematologic toxicity/disability | Facial nerve disorder, grade 2; Paresthesia, grade 3;  Peripheral motor neuropathy, grade 3;  Endocrine disorder, grade 3  (hypopituitarism) | no worsening |
| **Patient #5** | | |
| ECOG | 1-2 | 1-2 |
| Anemia | 0 | grade 2 |
| Thrombocytopenia | 0 | 0 |
| Leukocytopenia | 0 | 0 |
| Infections | 0 | Prostate infection, grade 3;  Anorectal infection, grade 3 |
| Non-hematologic toxicity/disability | Thromboembolic event (PE),  grade 3 | Urinary retention, grade 2 |
| **Patient #6** | | |
| ECOG | 0 | 1 |
| Anemia | grade 1 | grade 1 |
| Thrombocytopenia | grade 1 | 0 |
| Leukocytopenia | 0 | 0 |
| Infections | Peripheral nerve infection (VZV), grade 3;  Urinary tract infection, grade 3 | Pneumonia, grade 2 |
| Non-hematologic toxicity/disability | Ataxia, grade 2;  Cognitive disturbance, grade 2; Radiculitis, grade 3; Peripheral sensory neuropathy, grade 3; Facial nerve disorder, grade 2 | Spasticity, grade 1;  Remainig neurological symptoms after CAR-T completely resolved |
| **Patient #7** | | |
| ECOG | 4 | 4 |
| Anemia | grade 1 | grade 3 |
| Thrombocytopenia | 0 | grade 2 |
| Leukopenia | 0 | 0 |
| Infections | Urinary tract  Infection, grade 3 | Catheter related infection, grade 3 |
| Non-hematologic toxicity/disability | Seizure, grade 3  Adrenal insufficiency, grade 3 | Pancreatitis, grade 2;  Ileus, grade 2;  Alanine aminotransferase increase, grade 4 |
| **Patient #10** | | |
| ECOG | 1 | 1 |
| Anemia | grade 1 | grade 1 |
| Thrombocytopenia | 0 | grade 3 |
| Leukocytopenia | 0 | 0 |
| Infections | Enterocolitis, grade 3;  Urinary tract infection, grade 3 | Urinary tract infection, grade 3 |
| Non-hematologic toxicity/disability | Dysarthria, grade 1-2; Hyperglycemia, grade 3; | Hyponatremia, grade 1;  Alanine aminotransferase increase, grade 3;  Immune system disorders  (hemophagocytic lymphohistiocytosis; grade 5) |
| **Patient #11** | | |
| ECOG | 1 | 3-4 |
| Anemia | grade 1 | grade 2 |
| Thrombocytopenia | grade 1 | grade 4 |
| Leukocytopenia | 0 | grade 3 |
| Infections | Urinary tract infection, grade 3;  Pneumonia, grade 3;  Enterocolitis, grade 3 | Urinary tract infection, grade 3;  Peritoneal infection, grade 5 |
| Non-hematologic  Toxicity/disability | Vasculitis, grade 2;  Tremor, grade 3;  Amnesia, grade 1-2 | Stroke, grade 1  Ascites, grade 2  Rectal perforation, grade 5 |
| **Patient #13** | | |
| ECOG | 3 | 2 |
| Anemia | grade 2 | grade 2 |
| Thrombocytopenia | 0 | grade 4 |
| Leukocytopenia | 0 | 0 |
| Infections | Pneumonia, grade 3 | N/A |
| Non-hematologic toxicity/disability | Chronic kidney disease,  grade 1-2;  Ataxia, grade 2-3;  Vertigo, grade 2-3;  Amnesia, grade 2-3;  Vasculitis, grade 2;  Mitral valve disease, grade 3;  Heart failure, grade 2-3 | N/A |
| **Patient #14** | | |
| ECOG | 1 | 4 |
| Anemia | 0 | grade 4 |
| Thrombocytopenia | 0 | grade 4 |
| Leukocytopenia | 0 | grade 2 |
| Infections | 0 | Aspiration pneumonia, grade 3;  Cholecystitis, grade 2;  Sepsis, grade 5 |
| Non-hematologic toxicity/disability | Peripheral sensory neuropathy, grade 2;  Muscle weakness lower limb, grade 2;  Hyponatremia, grade 4;  Pleural effusion, grade 3;  Hyperparathyroidism, grade 2 | Colonic perforation, grade 4;  Ascites, grade 3; |
